# A novel speech analysis algorithm to detect cognitive impairment in a Spanish population

**DOI:** 10.3389/fneur.2024.1342907

**Published:** 2024-04-04

**Authors:** Alyssa N. Kaser, Laura H. Lacritz, Holly R. Winiarski, Peru Gabirondo, Jeff Schaffert, Alberto J. Coca, Javier Jiménez-Raboso, Tomas Rojo, Carla Zaldua, Iker Honorato, Dario Gallego, Emmanuel Rosario Nieves, Leslie D. Rosenstein, C. Munro Cullum

**Affiliations:** ^1^Department of Psychiatry, The University of Texas Southwestern Medical Center, Dallas, TX, United States; ^2^Department of Neurology, The University of Texas Southwestern Medical Center, Dallas, TX, United States; ^3^AcceXible Impacto, Sociedad Limitada, Bilbao, Spain; ^4^Cambridge Mathematics of Information in Healthcare Hub, University of Cambridge, Cambridge, United Kingdom; ^5^Parkland Health and Hospital System Behavioral Health Clinic, Dallas, TX, United States; ^6^Department of Neurological Surgery, The University of Texas Southwestern Medical Center, Dallas, TX, United States

**Keywords:** digital biomarkers, dementia, mild cognitive impairment, early detection, speech

## Abstract

**Objective:**

Early detection of cognitive impairment in the elderly is crucial for diagnosis and appropriate care. Brief, cost-effective cognitive screening instruments are needed to help identify individuals who require further evaluation. This study presents preliminary data on a new screening technology using automated voice recording analysis software in a Spanish population.

**Method:**

Data were collected from 174 Spanish-speaking individuals clinically diagnosed as cognitively normal (CN, *n* = 87) or impaired (mild cognitive impairment [MCI], *n* = 63; all-cause dementia, *n* = 24). Participants were recorded performing four common language tasks (Animal fluency, alternating fluency [sports and fruits], phonemic “F” fluency, and Cookie Theft Description). Recordings were processed via text-transcription and digital-signal processing techniques to capture neuropsychological variables and audio characteristics. A training sample of 122 subjects with similar demographics across groups was used to develop an algorithm to detect cognitive impairment. Speech and task features were used to develop five independent machine learning (ML) models to compute scores between 0 and 1, and a final algorithm was constructed using repeated cross-validation. A socio-demographically balanced subset of 52 participants was used to test the algorithm. Analysis of covariance (ANCOVA), covarying for demographic characteristics, was used to predict logistically-transformed algorithm scores.

**Results:**

Mean logit algorithm scores were significantly different across groups in the testing sample (*p* < 0.01). Comparisons of CN with impaired (MCI + dementia) and MCI groups using the final algorithm resulted in an AUC of 0.93/0.90, with overall accuracy of 88.4%/87.5%, sensitivity of 87.5/83.3, and specificity of 89.2/89.2, respectively.

**Conclusion:**

Findings provide initial support for the utility of this automated speech analysis algorithm as a screening tool for cognitive impairment in Spanish speakers. Additional study is needed to validate this technology in larger and more diverse clinical populations.

## Introduction

Alzheimer’s disease (AD) is the most common form of dementia and constitutes a significant public health problem worldwide due to the increasing need of social and economic services for patients and their support systems (1). A particular clinical challenge in AD and related dementias is identifying the early stages of cognitive decline due to its insidious onset and progression. AD is characterized by neuropathological features that can occur 15 to 20 years prior to noticeable changes in cognition or daily function (2, 3). Mild cognitive impairment (MCI) often represents a transitional stage between normal aging and dementia, and these individuals are at a greater risk of developing incident AD (4–8). Detecting early stages of cognitive decline is paramount for identifying those at greatest risk of progression to dementia and who may benefit from further evaluation and treatment (9). Improving the detection of early cognitive decline may lead to earlier initiation of disease modifying treatments to preserve independent functioning, which is crucial to a patient’s quality of life.

There currently exist numerous tools to help detect early clinical or pathological signs of MCI/dementia, including thorough clinical workups, neuropsychological evaluations, neuroimaging, and blood and cerebrospinal fluid biomarkers. While imaging and biomarker-based techniques can detect some neuropathological features associated with AD, some are invasive and not widely accessible for the purpose of early detection, and others are still in development. Formal neuropsychological evaluation is often an intrinsic part of the diagnostic process for detecting subtle changes in cognitive function in many centers. However, access to neuropsychological services may be limited due to various geographic, economic, and/or psychosocial factors, including limited access to healthcare resources, and therefore cannot be utilized effectively on a large scale. For these reasons, cognitive screening instruments are used as brief and cost-effective methods to identify individuals who require further evaluation and can be implemented across both primary care and specialized clinical settings. Primary care providers often serve as the first line of medical care for older adults concerned about their cognitive performance, yet physicians in primary care settings report feeling ill-equipped to recognize or document signs of cognitive decline (10, 11), contributing to gross under-detection of MCI in primary care settings (12). Nonetheless, current screening methods are not sufficiently sensitive or reliable in identifying clinical signs in the very early stages of dementia (13), and more precise screening techniques that can capture subtle changes in cognition are needed to identify individuals who may benefit from further evaluation.

Novel technologies and automated software systems to assess cognitive functioning in older individuals are emerging as new methods for early detection. In recent years, artificial intelligence (AI) and machine learning (ML) methods have surfaced as promising tools to aid in the early detection and diagnosis of AD and related disorders (14). More specifically, several studies have proposed the use of linguistic biomarkers for clinical classification and screening purposes (15–17). Identification and classification of language abnormalities play an important role in the diagnosis of AD, as subtle changes in various aspects of language have been identified in the early stages of the disease, including changes in number of between-utterance pauses (18), verbal fluency (19) and confrontation naming (20). Language impairment is typically defined by difficulties with word finding, comprehension, naming, and/or spontaneous speech. Simple tasks such as naming or verbal fluency can capture changes in language, and these markers of language impairment have been associated with early cognitive impairment in various neurodegenerative disorders and in some cases may precede other diagnostic clinical features (21, 22). However, these measures focus on the content of language rather than distinctive linguistic processes that may also be impacted in neurodegenerative disorders, such as speech rate, frequency and duration of pauses, and discourse efficiency (23). Furthermore, language tasks involve other cognitive processes that are relevant to the onset of dementia, including verbal working memory, attention, and processing speed (24). Therefore, sophisticated speech analyses might provide clinically relevant information, pertaining to both linguistic functions and different aspects of cognition (25). Moreover, the relative ease with which linguistic information can be accessed in a clinical context further favors the use of speech biomarkers as an accessible early detection tool.

In fact, machine learning (ML) techniques have been developed utilizing various speech and linguistic biomarkers to identify individuals with MCI (26), early AD (27, 28), dementia (29), Parkinson’s disease (30), and frontotemporal disorders (31). For instance, Hajjar and colleagues (27) found both acoustic and lexical-semantic biomarkers to be sensitive to cognitive impairment and disease progression in the early stages of AD. Several additional studies have demonstrated efficacy of computer-assisted linguistic processing algorithms in detecting speech differences between normal and impaired individuals in the English language (32, 33). Similar efficacy has been illustrated in other languages, including Chinese (34), Hungarian (26) and Swedish (35). While dementia-related linguistic changes in Spanish speakers continue to be an emerging area of research (36–38, 39), they remain understudied. Few investigations have explored automated speech analysis for detection of cognitive decline in a Spanish speaking sample (40, 41), and even so, published findings have limited interpretability for clinical use. Furthermore, several of these studies have been rated for high risk of bias due to sample selection (42), as measured by the QUADAS-2 checklist for quality assessment of diagnostic accuracy studies (43), potentially limiting its clinical applicability.

According to the Alzheimer’s Association, Hispanic individuals are disproportionately more likely to develop Alzheimer’s disease and related dementias (44) than their non-Hispanic White counterparts. Furthermore, this population is estimated to have the largest projected increase in ADRD over the next several decades (45). However, older adults in the Hispanic community frequently face challenges in receiving timely and accurate diagnoses. This is often attributed to the limited availability of bilingual providers and culturally validated neuropsychological tests, compounded by systemic barriers that hinder access to high-quality healthcare and specialized services (46). Survey findings revealed that Hispanic Americans rely on primary care or community health providers to screen for and address cognitive concerns, though standardized cognitive screening remains variable in such settings. Furthermore, in a scoping review of available cognitive screening tools for Spanish speaking populations, Burke and colleagues (47) suggested that some of the most frequently used cognitive screening tools may be less valid in this heterogenous population. As such, developing inclusive and culturally sensitive screening tools to detect cognitive impairment in this and other ethnic groups is crucial.

In the present study, we aimed to contribute to this area of exploration by presenting preliminary data on a novel cognitive screening technology that uses automated linguistic analysis software to quickly screen for MCI and dementia in a Spanish population. Furthermore, this study addresses a gap in the literature by introducing a brief, cost-effective, and readily available screening tool via web-based platform that is fully automated, highlighting its ease and accessibility in primary care and community health settings. The goals of the current manuscript were to (1) Validate a machine learning algorithm in a training sample, and (2) Test this algorithm in an aging Spanish-speaking testing sample to validate model performance when comparing cognitively normal (CN) with impaired (MCI + dementia) and MCI groups.

## Method

### Sample

Data were collected from 195 Spanish-speaking individuals recruited as part of a Multicenter Clinical Trial in Madrid, Getxo, and Quiron Salud, Spain. Participants were assessed by a neuropsychologist and neurologist and completed a series of cognitive tests as part of the initial evaluation: The Mini Mental State Examination (MMSE) (48), the 7-min screen test (49), Clock Drawing Test (50), Trail Making Test Parts A and B (51), the Blessed Dementia Scale (52), and four verbal tasks described below. Clinical diagnosis of normal cognition, MCI, or dementia was made by participating neurologists and neuropsychologists based on a full clinical work-up that included neurological and neuropsychological evaluation (without consideration of the speech processing variables). For a diagnosis of MCI, Petersen criteria (53) were utilized, and all subtypes of MCI were included (i.e., amnestic; multiple-domain; single-domain, non-amnestic). All-cause dementia was diagnosed in accordance with the DSM-IV criteria (54), requiring a notable decline in complex instrumental activities of daily living. Participants were considered eligible for the study if they (1) were proficient in the Spanish language, and (2) had been clinically diagnosed with either cognitive impairment (e.g., MCI or dementia) (7) or considered to have normal cognition. Exclusion criteria included prior diagnosis of a significant psychiatric disorder, cognitive impairment not due to a neurodegenerative process, and significant visual, hearing, or expressive language impairment that would impact the ability to complete cognitive tasks. Data from 21 participants were discarded due to incomplete cognitive data (*n* = 8), missing demographic information (*n* = 10), or poor text transcription quality on the audio recording used for algorithm modeling (*n* = 3). The sample was divided into a training set (*N* = 122), and a validating/testing set (*N* = 52; see Table 1 for sample characteristics).

**Table 1:** Demographic characteristics of the sample.

	Cognitively normal	Mild cognitive impairment	All-cause dementia	Impaired (MCI + Dementia)	*p*-value	Total
**Training sample**	*n* = 59	*n* = 51	*n* = 12	*n* = 63		*n* = 122
Age, years *M* (SD)	75.06 (8.14)	76.44 (8.08)	75.71 (5.35)	76.30 (7.64)	0.39^[1]^	75.71 (7.91)
Education, *M* (SD)	12.30 (5.40)	12.80 (5.40)	14.40 (4.70)	13.10 (5.20)	0.77^[1]^	12.20 (4.99)
Sex (% female)	58	49	25	44	0.20^[2]^	51.00
Clinical center (%)	34, 56, 1	29, 45, 25	75, 8, 17	38, 38, 24	0.06^[2]^	47, 36, 17
MMSE, *M* (SD)	28.58(1.32)	25.78(3.17)	23.33(1.97)	25.31(3.13)	<0.01^[1]^	26.90 (2.92)
**Test sample**	*n* = 28	*n* = 12	*n* = 12	*n* = 24		*n* = 52
Age, years *M* (SD)	65.54 (5.98)	77.09 (6.28)	73.12 (9.87)	75.11 (8.51)	<0.01^[1]^	70.04 (8.71)
Education, *M* (SD)	12.75 (3.47)	13.50 (4.54)	13.17 (4.34)	13.33 (4.44)	0.61^[1]^	13.02 (3.96)
Sex (% female)	89	42	5	46	<0.01^[2]^	69
Clinical center (%)	54, 32, 14	58, 25, 17	92, 0, 8	75, 12, 12	0.21^[2]^	63, 23, 13
MMSE, *M* (SD)	28.32 (1.58)	26.33 (2.78)	22.08 (1.89)	24.21 (3.19)	<0.01^[1]^	26.42 (3.20)

^[1]^: two sample (Welch’s) *t*-tests and ^[2]^: Chi-square test for proportions were computed between cognitively normal and impaired (MCI + Dementia) groups. Clinical centers included Getxo, Madrid, and Quiron, respectively.

### Measures

#### Cognitive assessments

**Figure 1:**
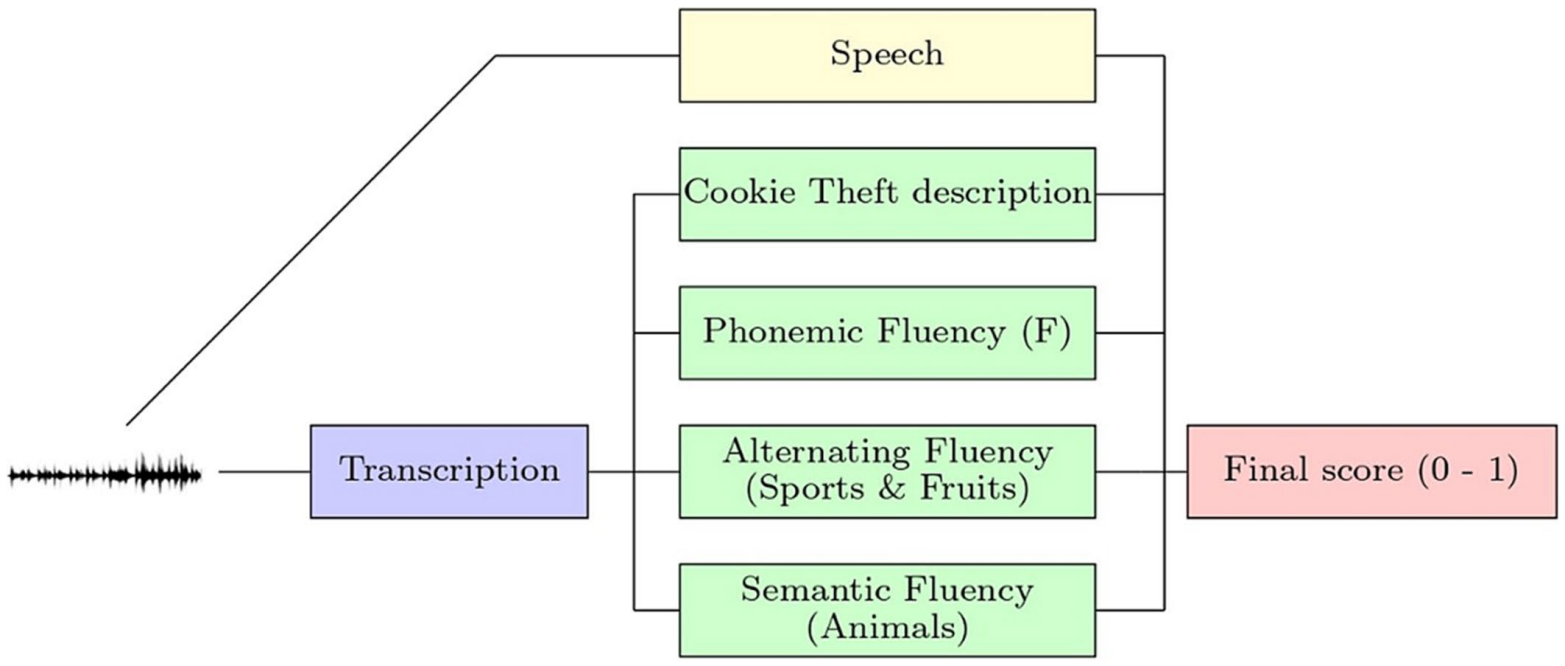
Five classifiers combined to create machine learning algorithm.

The current study utilized four language tasks for capturing neuropsychological performance and audio recording variables for each participant, including (1) Picture Description Task (PDT), using the “Cookie Theft” picture from the Boston Diagnostic Aphasia Examination which assesses spoken language (55); (2) Phonemic Verbal Fluency (PVF), measured by the letter (F) word generation task; (3) Alternating Verbal Fluency (SVF-Alt), measured by alternating fruits and sports word generation task; and (4) Semantic Verbal Fluency (SVF), measured by animal naming fluency (see Figure 1). Tasks were administered and audio files were recorded and stored through the AcceXible platform, a proprietary instrument developed for disease detection through speech analysis. AcceXible is a web-based platform that can be accessed via web browser or mobile application designed for Apple® devices, and utilizes cloud-based data processing and storage features (56; see Supplementary Figure S1). Once the initial task was initiated by the clinician or administrator, participants followed instructions presented on the computer interface for each of the four tasks. Total administration time was approximately 5 min.

### Acoustic and linguistic feature extraction

Recordings were processed via speech-to-text transcription (Google Cloud’s Speech-to-Text) and digital-signal processing techniques to capture neuropsychological variables and audio characteristics, respectively. SVF, SVF-Alt and PVF tasks were restricted to 1 min each to ensure that all audio files had a uniform duration of 1 min (mean length of ~61 s). Due to the nature of the task, the Picture Description Task (PDT) was not limited based on time, and audio recording concluded based on provider’s clinical judgment. Audio recordings were segmented into 25 ms windows, a practice commonly seen in audio segmentation literature (Supplementary Table S1) (57). Audio segments were then preprocessed by normalizing the amplitude, to minimize bias toward signals with higher or lower energy levels, and using Butterworth low-pass filter, to attenuate high frequencies and eliminate impact of environmental noise (58, 59). Variables were computed and used for voice model training using the Python library “librosa” (60). Extracted variables from verbal tasks included consecutive repetitions, mean duration and standard deviation of silences, number of repeated words, and other task-specific variables. An exhaustive list of extracted speech features and verbal task variables used for model training can be found in Tables 2, 3.

**Table 2:** Verbal task variables extracted for model training.

Variable	Variable description	Verbal task
Mean repetitions	Total and consecutive repeated animals divided by the number of listed animals	SVF
Consecutive repetitions	Number of consecutive repetitions divided by the number of words	SVF
Number semantic clusters	Number of semantic clusters. Cluster is defined as a group of animal words consisting of successively generated words belonging to the same subcategory	SVF
Average cluster size	Average number of animals in semantic clusters	SVF
Average switches	The number of switches in semantic clusters divided by the total number of animals	SVF
Not animals	Number of words that are not animals, divided by the total number of listed words	SVF
Mean silence	Mean duration of silences	SVF, SVF-Alt, PVF, PDT
Std. silence	Standard deviation of duration of silences	SVF, SVF-Alt, PVF, PDT
Number of temporal clusters	Number of temporal clusters or “spurts” of animal words	SVF
Average cluster size	Average number of words in temporal clusters	SVF
Count sports/fruits	Total number of sports/fruits said by the participant	SVF-Alt
Not alternate	Total number of words out of alternate sequence	SVF-Alt
Not in list	Total number of words that are neither fruits nor sports	SVF-Alt
Number of words	Total number of animals/words that start with “F”; Total number of words	SVF, PVF
Total repetitions	Total number of repeated words	PVF
Count consonant	Proportion of consonant letters in words that start with “F”	PVF
Vocabulary size	Number of unique lemmas said by the patient	PDT
Percentage of parts of speech	Number of verbs/nouns/pronouns/determinants/adjectives, divided by the number of words	PDT
Number of words 30″	Number of words said in the first 30 s	PDT
Number of keywords	Number of detected keywords. List of keywords formed by the main parts of image	PDT

SVF, Semantic Verbal Fluency; SVF-Alt, Alternate Semantic Verbal Fluency; PVF, Phonemic Verbal Fluency; PDT, Picture Description – Cookie Theft.

**Table 3:** Speech Feature Variables Extracted for Model Training.

Variable	Variable description	Model task
Chromagram	Represents the tonality of the voice using a vector of 12 parameters	Speech
MEL	Represents the spectral information of a voice signal using a vector of 13 parameters	Speech
RMS (Root Mean Square)	Represents the average amplitude of the voice signal (volume)	Speech
ZCRS (Zero Crossing Rates)	The number of times the voice signal crosses the horizontal axis (zero) during a specific time period, normalized by the duration of that time period.	Speech
Tempo	The frequency of rhythmic pulses in the audio signal, measured in beats/min (speed)	Speech
OENV (Onset Envelope)	Represents the audio signal that highlights rapid changes in amplitude, also known as onsets. Onsets refer to abrupt changes in the energy level of an audio signal, which can indicate the beginning of a musical note or a syllable in spoken voice.	Speech
Spectral bandwidth	Represents the frequency bandwidth that contains a certain fraction of the total spectral energy of the audio signal.	Speech
Spectral bandwidth	Represents the center of the frequency spectrum of a voice signal.	Speech

Variables extracted using the Python library librosa.

### Algorithm development and model training

Clinical diagnosis served as the target variable for training the ML model. In the current study, this variable contained three diagnostic categories: CN, MCI, and dementia. The model considered the objective variable as dichotomous, with a score of zero indicating cognitively normal and a score of one indicating impairment (MCI + dementia or MCI, depending on the impairment group of interest). The model’s objective was to identify the earliest stages of cognitive decline, as well as overt dementia. As previously mentioned, the initial dataset was separated into a training and testing set using a 70%/30% split to ensure a socio-demographically balanced training sample. The training procedure was applied to the training set and the testing set was used to test the final model. To train a model that had the same predictive power when including sociodemographic information, a demographically similar subsample (age, sex, education, and clinical center) across groups of diagnoses was used.

First, the linguistic and acoustic features were extracted and preprocessed using text transcription techniques and digital-signal preprocessing, respectively. In the case of acoustic features (see Table 3), a feature selection step was added in each iteration of the Cross Validation due to the high number of features. The feature selection was based on the F-statistic value obtained in the analysis of variance. The number of selected features was added as a hyperparameter in the Grid Search CV, so that the best “k” features were selected based on F-statistic results, given a grid (5, 10, 15, 20). In other cases (e.g., SVF, SVF-Alt, PDT, and PVF), all computed features described in Table 2 were used to train the ML models. Features were standardized before building the models. The detailed descriptions of these variables are presented in Tables 2, 3. For each variable described in Table 2, including neuropsychological variables, a Python-based function was defined to perform its extraction and calculation, allowing for automatic computation when the transcription was carried out (61–63). All predictive models used in this study were classic ML models registered in scikit-learn software (64). These models include Logistic Regression, Supported Vector Machines, K-Nearest Neighbors, Random Forest and Gradient Boosting. The strategy used to build models was Grid Search 10-fold Cross Validation (CV), where model selection was performed by maximizing AUC score (Supplementary Data).

**Figure 2:**
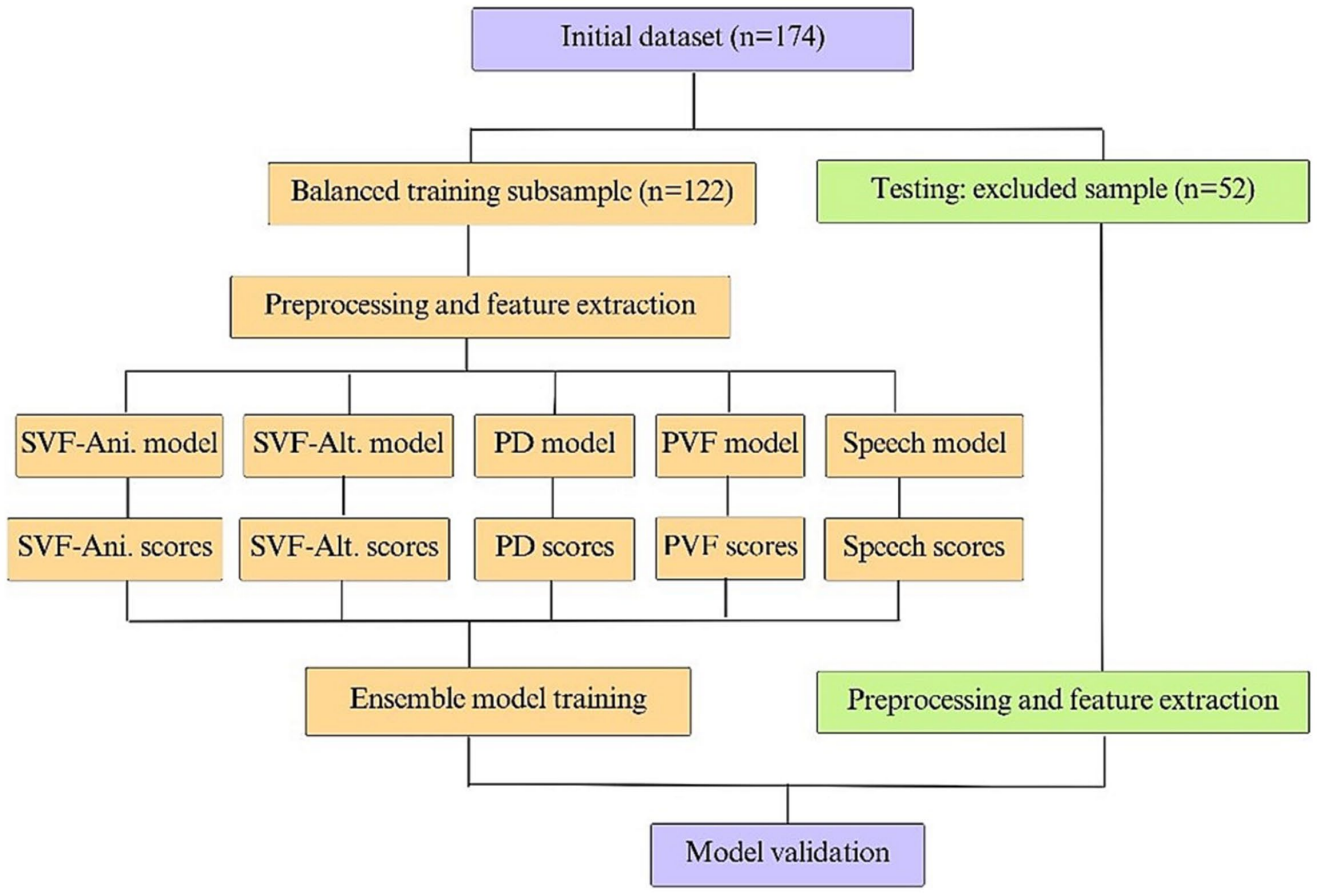
Model training and testing scheme.

Subsequently, five independent ML models were trained: one for each task, using the extracted linguistic variables and another one with the digital-signal processing features, as demonstrated in Figure 2. When building the model based on acoustic features, a Support Vector Machine (SVM) with a linear kernel and regularization strength of 10 was used. A feature selection step was incorporated to address the high number of acoustic features, with the optimal number of features determined to be 15 by the SVM (see Supplementary Table S2). Each one of the five independent models followed the same training procedure. A 10-fold CV was performed 10 times to increase robustness and avoid overfitting. The algorithm development procedure began by first defining a list of candidate ML classical models and identifying their respective hyperparameter grids. For each model candidate, the sample was randomly stratified into a 90/10 split, a commonly used train/test ratio when implementing a 10-fold CV procedure (65). Next, model parameters were calculated on the training set and scores were calculated for the new testing set, ranging from zero to one. Sensitivity, specificity, accuracy, area under the curve (AUC), and mean squared error (MSE) were calculated for each possible cutoff point from zero to one. These steps were repeated 10 times and the mean of each calculated metric was reported, at which point this step was completed 10 more times to report the mean and variance of each obtained result. The model and cutoff point that demonstrated superior performance based on all calculated metrics was then manually selected. Finally, the parameters of the selected model were calculated for the training set sample. The threshold was chosen during the training phase. For each candidate threshold (ranging from 0 to 1 in increments of 0.01), we computed the mean accuracy, mean sensitivity, and mean specificity using a 10-fold CV approach. The optimal threshold point for detecting impaired (MCI + dementia) and MCI groups was determined by (1) maximizing the sum of mean sensitivity and mean specificity by way of Youden’s J statistic (66) [calculated as (sensitivity + specificity – 1)] and (2) ensuring that the mean sensitivity exceeded the mean specificity at that point. If multiple threshold points met these conditions, the smallest threshold value was selected to prioritize sensitivity over specificity.

Once this procedure was performed for the various models (see Figures 1, 2), the predicted scores were computed for each model (i.e., four tasks and one speech) to create five individual scores for each participant. Finally, an ensemble ML model was trained in which the input included the five individual scores, and the output was one unique score. Before training the ensemble model, logit transformation was applied to each obtained score to create a continuous value in the range of negative infinity to infinity to obtain a final model.

### Statistical analyses

Demographic characteristics for the training and testing samples are presented in Table 1. Comparisons between cognitively intact and impaired groups were calculated using two sample t-test in case of continuous variables (i.e., age, education, MMSE) and chi-square test for proportions in case of categorical variables (i.e., sex, clinical center). General linear modeling was used to test whether age, sex, and education significantly predicted logistically transformed algorithm scores in the training and testing sets. Receiver operating characteristic (ROC) curves were constructed for the testing set when discriminating CN and impaired (MCI + dementia) or MCI groups, and areas under the curves (AUC) were used as measures of overall classification accuracy. Accuracy, sensitivity, specificity, and F-scores were calculated for various threshold values for impaired and MCI groups. F-scores represent a common machine learning metric used to measure model accuracy by way of balancing precision and recall, with precision representing the proportion of correct “positive” predictions made by the model, or positive predictive value, and recall representing the proportion of actual positive samples correctly identified by the model, or sensitivity (67). An F-score value ranges from 0 to 1, with a 1 indicating perfect precision and recall. ANCOVA was used to examine differences in mean logit and algorithm scores by CN, MCI, and all-cause dementia groups, controlling for age, sex, and education. Before performing this test, logit transformation was applied to better meet the assumptions of the test, since the outcome variable might be the prediction of a non-linear ML model, and residuals might show a non-constant spread. Finally, we computed accuracy, sensitivity, and specificity across different MMSE score thresholds for both impaired and MCI groups to compare the classification accuracy with that of the presented model. Statistical analyses were conducted using the *statsmodels* and *pingouin* packages in Python.

## Results

### Descriptive statistics

Demographic and clinical data of the 174 participants are described in Table 1. The cohort had a mean age of 74.03 years (SD = 8.55), completed an average of 12.45 years of education (SD = 4.72), and included slightly more females than males (56%). Demographic characteristics were similar across groups in the training set, but age and sex were significantly different between clinical groups in the testing sample. MMSE scores were different across groups in both samples (Supplementary Figure S2). A one-way ANCOVA, controlling for age, sex, and years of education, demonstrated a significant difference in mean logit and algorithm scores for every independent model’s prediction across CN, MCI, and all-cause dementia groups, except for phonemic verbal fluency (see Table 4).

**Table 4:** Mean logit and algorithm scores by clinical diagnosis in the testing sample.

	Cognitively normal	Mild cognitive impairment	All-cause dementia	Impaired (MCI + Dementia)	*p*-value, F
**Logit score, *M* (SD)**	*n* = 28	*n* = 12	*n* = 12	*n* = 24	–
Semantic verbal fluency	−0.99 (0.83)	0.55 (1.01)	1.65 (0.86)	1.10 (1.09)	<0.01, 18.97
Semantic verbal fluency-alternating	−0.46 (0.57)	−0.06 (0.65)	0.14 (0.46)	0.04 (0.57)	<0.01, 15.41
Picture description task	−0.22 (0.22)	−0.06 (0.30)	0.28 (0.32)	0.11 (0.36)	<0.01, 7.99
Phonemic verbal fluency	−0.25 (0.40)	0.18 (0.32)	0.69 (0.39)	0.43 (0.44)	0.06, 2.84
Speech	−0.10 (0.48)	−0.04 (0.64)	0.45 (0.59)	0.20 (0.66)	0.02, 4.20
Final score	−2.83 (2.81)	2.29 (2.38)	3.39 (1.94)	2.84 (2.24)	<0.01, 15.78
**Algorithm score, *M* (SD)**	*n* = 28	*n* = 12	*n* = 12	*n* = 24	–
Semantic verbal fluency	0.29 (0.16)	0.62 (0.21)	0.81 (0.14)	0.71 (0.20)	<0.01, 21.14
Semantic verbal fluency-alternating	0.39 (0.13)	0.49 (0.15)	0.53 (0.11)	0.51 (0.13)	<0.01, 15.36
Picture description task	0.45 (0.05)	0.49 (0.07)	0.57 (0.08)	0.53 (0.09)	<0.01, 8.01
Phonemic verbal fluency	0.44 (0.10)	0.54 (0.08)	0.66 (0.09)	0.60 (0.10)	0.06, 2.93
Speech	0.48 (0.11)	0.49 (0.15)	0.60 (0.13)	0.55 (0.15)	0.02, 4.13
Final score	0.14 (0.29)	0.79 (0.32)	0.90 (0.21)	0.85 (0.27)	<0.01, 22.56

ANCOVA compared differences in mean logit and algorithm scores across cognitively normal, MCI, and all-cause dementia groups, controlling for age, sex, and years of education.

### Model performance

**Figure 3:**
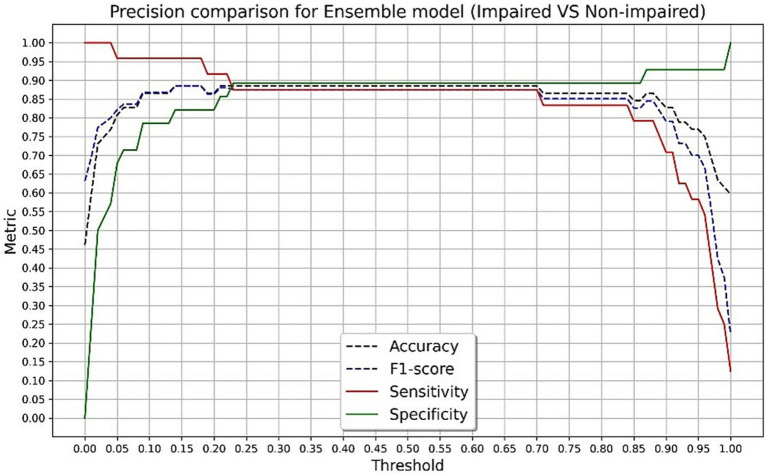
Accuracy, F-score, sensitivity and specificity for each threshold value in discriminating cognitively normal and impaired groups in the testing set.

**Figure 4:**
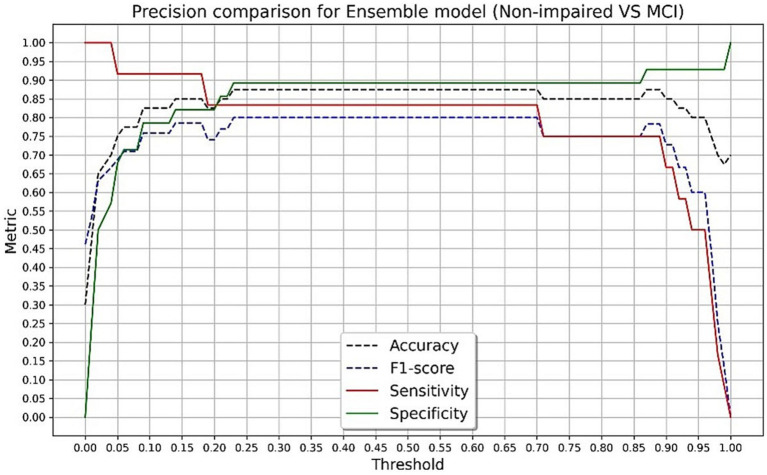
Accuracy, F-score, sensitivity and specificity for each threshold value in discriminating cognitively normal versus mild cognitive impairment groups in the testing set.

The final algorithm in the testing sample with the presented tool resulted in an AUC of 0.93 with an overall accuracy of 88.4%, sensitivity of 87.5%, specificity of 89.2%, and F-score of 0.87 in discriminating CN and impaired groups. The final algorithm in the testing sample obtained an AUC of 0.90 with an overall accuracy of 87.5%, sensitivity of 83.3%, specificity of 89.2%, and F-score of 0.80 in discriminating CN and MCI groups. Using Youden’s Index, the optimal threshold value was determined to be 0.45 for impaired (*J* = 0.767) and MCI (*J* = 0.725) groups. Figures 3, 4 present accuracy, F-score, sensitivity and specificity for each threshold value when discriminating CN versus impaired and MCI groups in the whole testing set. Since the selected optimal ensemble ML model is RandomForest, the distribution of scores was non-normal and scores fell close to 0 or 1. Therefore, metrics remained constant between the thresholds of 0.23 and 0.70. The mean squared error obtained in the testing set was 0.10 when distinguishing impaired from non-impaired and 0.114 when distinguishing MCI from non-impaired.

Table 5 presents the performance of each machine learning model in the testing sample. Accuracy, sensitivity, and specificity were identical across SVF and ensemble models. Nonetheless, the final ensemble model demonstrated the best overall performance (AUC = 0.93) compared to each individual ML model, with the speech-only model performing the worst (AUC = 0.64). For comparison, when differentiating impaired from non-impaired groups, the MMSE demonstrated an accuracy range of 71.1 to 80.7%, sensitivity range of 41.6 to 66.6%, and specificity range of 75 to 100% across cut-offs of 24 to 28, with an optimal cut-off of 27 yielding an accuracy of 76.9%, sensitivity of 66.6%, and specificity of 85.7%. When differentiating MCI from non-impaired, the MMSE demonstrated an accuracy range of 62.5 to 77.5%, sensitivity range of 8.3 to 33.3%, and specificity range of 75 to 100% across cut-offs of 24 to 28, with an optimal cut-off of 27 yielding an accuracy of 70%, sensitivity of 33.3%, and specificity of 85.7%.

**Table 5:** Machine learning models’ performance in the testing sample for cognitively normal versus impaired and MCI groups.

	AUC	Accuracy	Sensitivity	Specificity
Semantic verbal fluency – animals	0.91/0.85	88.40/87.50	87.50/83.3	89.20/89.20
Semantic verbal fluency - alternating	0.87/0.79	78.70/72.50	87.50/75.00	71.40/71.40
Picture description	0.76/0.64	69.20/65.00	79.00/58.30	67.80/67.80
Phonemic verbal fluency	0.73/0.68	65.30/62.50	70.80/66.60	60.70/60.70
Speech	0.64/0.52	59.60/55.00	58.30/41.60	60.70/60.70
Final ensemble	0.93/0.90	88.40/87.50	87.50/83.30	89.20/89.20

Results are obtained in the testing set, using CN versus Impaired / CN versus MCI groups, respectively. Model training derived from verbal task and speech feature variables outlined in Tables 2, 3.

## Discussion

Novel automated technologies are emerging to allow methods to screen for subtle cognitive changes in older adults, as early detection is critical for diagnosis and initiation of appropriate care. This is especially important for historically marginalized racial and ethnic groups, who often experience delayed diagnosis of cognitive disorders and face barriers in accessing specialists for evaluation and treatment. Furthermore, these evolving technologies hold promise as brief, accessible, and scalable means of capturing emerging cognitive deficits. The current study explored the utility of an automated speech analysis algorithm to quickly and effectively screen for cognitive impairment in a Spanish speaking population.

### Model performance

**Figure 5:**
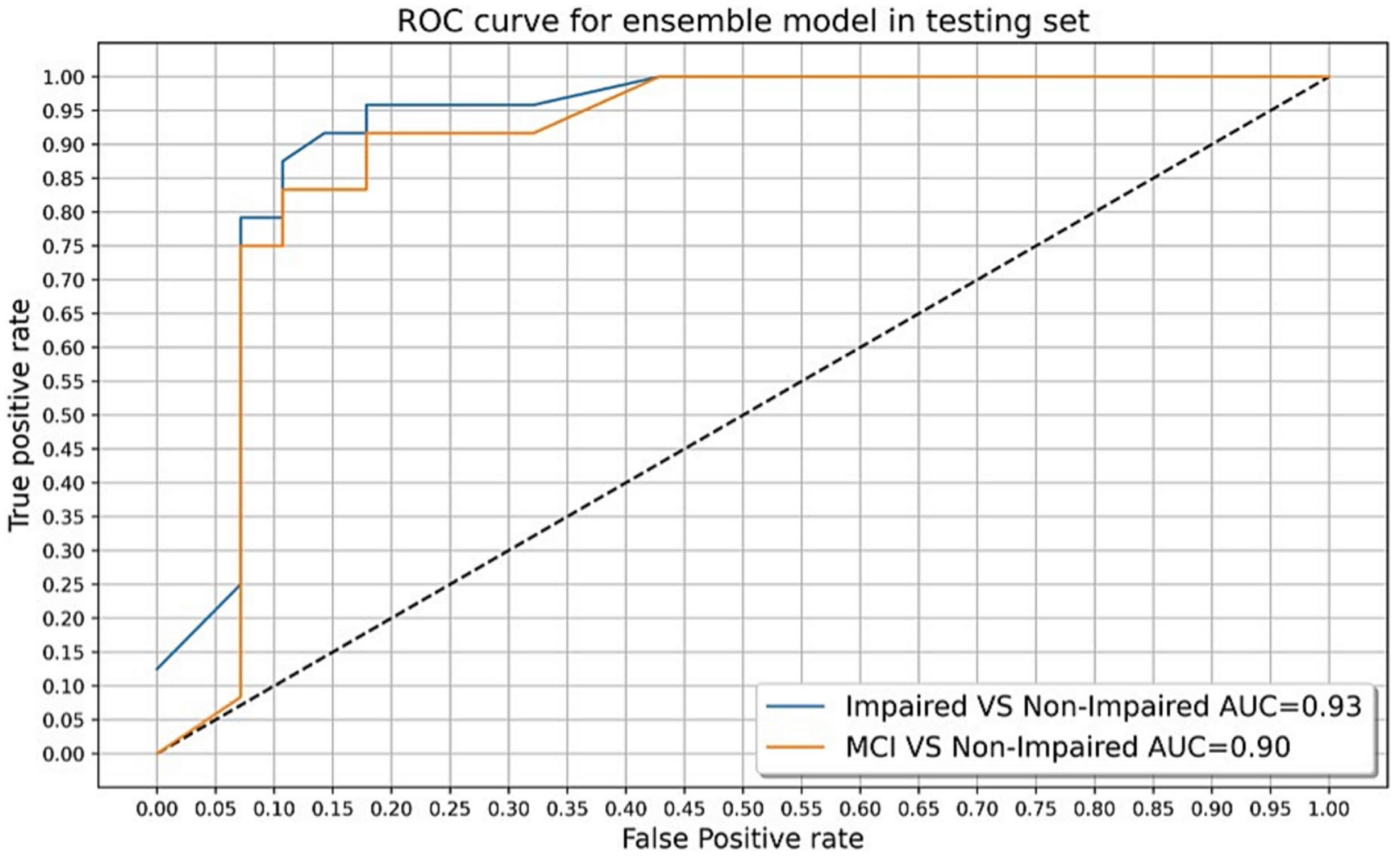
Receiver operating characteristic (ROC) curves and associated AUC values for cognitively normal (CN) versus mild cognitive impairment group, and CN versus impaired (MCI + dementia) group in the testing set.

This speech analysis algorithm was able to accurately differentiate CN from impaired (MCI + dementia) and MCI groups with an overall accuracy of 88.4 and 87.5% in the testing set, respectively (Table 5). Examination of the associated AUC values of CN versus impaired and MCI groups in Figure 5 reveals close similarities between classification curves, both falling in the outstanding range (0.93 for impaired and 0.90 for MCI). Furthermore, F-scores for the final algorithm in both impaired and MCI groups were considered good, indicating that both precision and recall of the ML model were high. Therefore, this automated speech analysis algorithm holds promise for distinguishing both early and more advanced cognitive decline from those who are cognitively normal in the studied sample. Prior efforts toward developing speech analysis tools for early detection of cognitive impairment have demonstrated similar differentiating abilities, with initial results exhibiting high accuracy (80–95%) in the detection of subtle changes in speech of those diagnosed with MCI and AD (42). Previous ML studies have demonstrated promising diagnostic ability when using similar verbal tasks in various languages, including spontaneous speech (68), picture description task (69), and fluency tasks (70), in combination with acoustic and linguistic features of speech. The current approach adds to the literature by utilizing numerous verbal tasks in one ML model for a Spanish-speaking population, while simultaneously maintaining a brief administration time, though future research may clarify which task and/or tasks contribute most to the success of the model. For instance, given that the semantic verbal fluency model with associated verbal characteristics performed similarly to the final ensemble model in the current study, abbreviated versions of this task with similar screening abilities may be possible and should be further explored.

### Strengths, limitations, and future directions

The current study provides support for the utility of a quick, 5-minute assessment that analyzes responses to language measures to investigate aspects of speech that may not be easily detectable to examiners. Early screening and detection of cognitive impairment in at-risk populations may allow for the proper allocation of time and resources to individuals identified as requiring more extensive assessment (e.g., biomarker analysis, comprehensive neuropsychological evaluation, neuroimaging, etc.). Additionally, this speech analysis tool may outperform current standard-of-care screening measures such as the Mini-Mental State Examination. In this study, the technology outperformed the MMSE in screening for MCI and dementia among Spanish speakers. The tool demonstrated superior accuracy (87.5/88.4), sensitivity (83.3%/87.5%), and specificity (89.2%/89.2%) when compared to an MMSE cut-off of 24 or 25, two thresholds well-established in clinical practice (71). While qualitatively different, the current model’s performance seems comparable, if not superior, to the MMSE.

Blesa and colleagues developed age and education-adjusted cut-off scores for the MMSE in a Spanish speaking cohort, identifying the optimal cut-off score to be 24/25 which yielded a sensitivity of 87.32% and specificity of 89.19% in detecting dementia (72). Similar results have been demonstrated using the Montreal Cognitive Assessment (MoCA) in the detection of MCI among Spanish speakers, with a sensitivity of 80.48% and specificity of 81.19% (73). Beyond its superior sensitivity, this technology fills a gap in the existing literature as a concise, cost-effective screening tool that is available through a web-based platform. The tool is fully automated, partially addressing issues related to the frequent incongruence between the patient and examiner’s native language, and ultimately emphasizing its ease and accessibility in primary care and community health settings. Furthermore, the impact of the current model is a promising avenue to increase cognitive screening, as it could be easily adapted to mobile phones, the feasibility and efficacy of which has been demonstrated in prior studies (74, 75). Thus, the current technology may allow for screening of more individuals that may be otherwise unable to access care.

Despite the promising findings for advancing the detection and monitoring of cognitive impairment, certain limitations must be noted. First, the current state of machine learning research emphasizes the need for standardization of training set size determination. Future studies would benefit from an increased sample size to improve robustness and minimize bias of the clinical model. Second, speech analysis software relies on high-quality audio recording and transcription to ensure accurate interpretation of speech. This study experienced varied audio-quality during data collection, requiring three cases with poor quality to be excluded from analyses. Nonetheless, speech-to-text transcription models should continue to be updated with technological advances to further improve efficacy in future machine learning and technology studies. Third, this study included only Spanish speaking individuals from selected sites in Spain, and thus, the generalizability of the algorithm in other populations will require additional validation. To extend the utility of the current technology, future research may focus on other Spanish-speaking populations from various geographic locations which could represent dialectical differences. To this point, preliminary data utilizing this technology with a U.S.-based sample of Spanish speakers demonstrated the impact subtle variations in Spanish expressive language may have on accuracy of transcriptions (76). However, modification of the transcription tool to include these language variations resulted in an overall transcription accuracy of 95%. Therefore, differences in cultural exposure and regional dialect should be considered in the development and use of automated speech software.

While certain aspects of the current model are inherently more naturalistic than simple quantitative assessments of speech production, it can be argued that elements of the task are not entirely naturalistic (e.g., listing animals). Furthermore, the speech samples are somewhat constrained by the assessment setting, and thus may not accurately reflect an examinee’s naturalistic speech qualities. Various forms of spontaneous speech have shown to be sensitive to cognitive decline, including analysis of informal conversations with others (77) and detailed recollections of the preceding day’s events (78) or patients’ daily routine (79). Therefore, further research would be helpful in determining how the presented model’s assessment of these variables compares between the test setting and a more natural speech interaction, and how that might influence clinical interpretation.

Though speech analysis technology would provide an affordable and accessible option for identification of cognitive impairment, it is critical that investigations integrate biomarkers, more detailed neuropsychological evaluations, and neuroimaging findings to demonstrate incremental validity and increase our confidence in the clinical models. Additionally, as promising as the current model appears, future research may focus on the utility of the tool in predicting those at risk of conversion from MCI to dementia. For example, current stand-alone single-administration screening tools such as the MMSE are not effective for this use and may depend upon serial assessments, ideally with more comprehensive evaluations (80). Similarly, the tool may be further validated for use in differential diagnosis of neurodegenerative and other neurological conditions. A critical review on the role of connected speech in neurodegenerative diseases revealed numerous studies highlighting unique speech features in clinical populations, including amnestic MCI, primary progressive aphasia, movement disorders, dementia due to Alzheimer’s disease, and amyotrophic lateral sclerosis (23). Such detailed assessments of speech samples may add further value to traditional language assessment instruments.

## Conclusion

The current findings provide initial support for the utility of an automated speech analysis algorithm to quickly and efficiently detect cognitive impairment in an older Spanish-speaking population. Results suggest that the algorithm has a robust ability to detect early stages of cognitive decline and clear dementia. Further research is needed to validate this methodology in additional languages and clinical populations, as this may be a valuable cross-cultural screening method for MCI and dementia.

## Data availability statement

The raw data supporting the conclusions of this article will be made available by the authors, without undue reservation.

## Ethics statement

The studies involving humans were approved by the CEIC Hospital Clínico San Carlos. The studies were conducted in accordance with the local legislation and institutional requirements. The participants provided their written informed consent to participate in this study.

## Author contributions

AK: Writing – original draft. LL: Writing – review & editing. HW: Writing – original draft. PG: Writing – original draft. JS: Writing – review & editing. AC: Writing - original draft. JJ-R: Writing – review & editing. TR: Writing – review & editing. CZ: Writing – review & editing. IH: Writing – review & editing. DG: Writing – review & editing. EN: Writing – review & editing. LR: Writing – review & editing. CC: Writing – review & editing.
