# Supplementary material for: A novel speech analysis algorithm to detect cognitive impairment in a Spanish population
Source: Front Neurol. 2024 Apr 4;15:1342907. doi: 10.3389/fneur.2024.1342907 (PMC11024431; doi:10.3389/fneur.2024.1342907)
Supplement: Supplementary file 1 [file Data_Sheet_1.PDF]

Supplementary Material

A Novel Speech Analysis Algorithm to Detect Cognitive Impairment in a Spanish Population

1 Supplementary Data

Model Architecture and Training

Neither CNNs, LSTMs, nor transformers were used in our model architecture or training. The hyperparameter tuning strategy used was Grid Search 10-fold Cross Validation, where the maximized metric was AUC. The classic ML models that were included in the grid with its hyperparameter values were the Logistic Regression, Supported Vector Machines (SVMs), K-Nearest Neighbors, Random Forest and Gradient Boosting, all implemented in the well-known open-source scikit-learn library (Buitinck et al., 2013). All data splits inside the Cross Validation loop were balanced with respect to the impaired and non-impaired participant proportion.

Grid Search 10-fold Cross Validation was implemented to select the optimal ML model and its hyperparameters. The model and its hyperparameters were selected by maximizing the AUC score, and parameters or weights were fitted using the entire training set. This procedure was repeated for each computed feature set (i.e., the five trained independent ML models): acoustic features obtained from the whole audio recording and lexical-semantic features obtained from SVF Animals, SVF-Alternating, PDT, and PVF. In the case of the acoustic features, a feature selection step was added in each iteration in the Cross Validation due to the high dimensionality. The feature selection was based on the F-statistic value obtained in the analysis of variance. The number of selected features was added as a hyperparameter in the Grid Search Cross Validation, so that the best "k" features were selected, based on t-test results. In other cases, all computed features described in Table 2 were used to train the ML models. Features were standardized before building the models.

Once the five independent models were selected and fitted, a final ensemble model was built. For this, the prediction (i.e., estimated probability for being classified in the impaired group) from each of the five independent models was generated and transformed using the logit function to achieve linearity and stabilize the estimated probabilities. After obtaining five logit-scores for each participant in the training set, the Grid Search 10-fold Cross Validation procedure was once again applied to train the ensemble model. Here, the five logit-scores were treated as five independent predictive features. The reviewer brings up a good point about verifying that the models did not overfit the data. In this case, it is possible that the ensemble model may be overfitted inside the Grid Search Cross Validation loop. This is because the logit-scores in the validation set optimally maximize the AUC. However, the final reported error and model's evaluation is performed in the testing set and is not included in any step of the training procedure. Therefore, this ensures that it is free from overfitting.

Hyperparameter Tuning for the Machine Learning Model

- Logistic Regression:
    - C (inverse of regularization strength): [0.1, 0.5, 1, 2, 10, 1000, 10000]
    - Penalty: L1 or L2
- Supported Vector Machines:
    - C (inverse of regularization strength): [0.1, 0.2, 0.5, 1, 2, 5, 10]
    - Estimated kernel: Radial Basis Function kernel or linear kernel.
- K-Nearest Neighbors:
    - Number of neighbors to consider: [2, 4, 6, 8, 10, 12, 14, 16, 18, 20, 22, 24, 26, 28, 30]
- Random Forest:
    - Whether bootstrap samples are used when building trees: yes or no
    - The maximum depth of the tree: [10, 20, 30]
    - The minimum number of samples required to be at a leaf node: [1, 2, 4]
    - Number of trees: [100, 200]
- Gradient Boosting:
    - Learning rate or the contribution of each tree: [0.01, 0.1, 0.15]
    - The minimum number of samples required to split an internal node: [0.1, 0.2, 0.3, 0.4]
    - Maximum depth of the individual regression estimators: [3, 5, 8]
    - The number of features to consider when looking for the best split: log2(original number of features) or sqrt(original number of features)
    - The function to measure the quality of a split: mean squared error with improvement score by Friedman, or just the mean squared error.

The fraction of samples to be used for fitting the individual base learners: [0.5, 0.9, 1.0]

AcceXible Platform Architecture

The entire AcceXible system code can be divided into three modules: front-end or interface code, API or back-end, and storage of data and ML models. The front-end code is responsible for presenting content and interacting with users, as well as sending information to the back-end module. The back-end module handles internal logic and implements various modules within the software, including database management, user authentication, audio tokenization, and sending results to users. Data storage is organized by three databases: user administration, audio storage, and data generated during the process (e.g., transcription). Microsoft Azure SQL database is used for user administration, Microsoft Azure Storage is used for audio storage and Microsoft Azure Cosmos for model output data storage. Finally, ML models are saved in the open-source platform MLflow, which facilitates experimentation, reproduction and implementation of ML models in production environments. This platform records metrics, training data and characteristics of each model.

2 Supplementary Tables

Supplementary Table 1. Descriptive Statistics of Audio Segments for ML Training and Testing Sets

Task	Min	Max	Stand. Dev.	Mean
Training Set				
SVF-Animals	60.07	65.9	2.01	60.98
SVF-Alternating	60.07	65.9	1.98	60.96
PVF	60.07	100.12	4.08	61.38
PDT	20.13	217.36	28.98	76.61
Testing Set				
SVF-Animals	60.07	65.9	1.88	60.83
SVF-Alternating	60.07	65.87	1.68	60.67
PVF	59.0	66.0	1.83	60.73
PDT	30.39	186.27	27.82	69.53

Note. Values reflect the duration of audio segment in seconds.

Supplementary Table 2. Acoustic Features Included in Model

Selected feature	Weight in SVM model
Mean of frequency bandwidth	-14.79
Variance of MEL's sixth parameter	-13.63
Mean center of the frequency spectrum	9.85
Variance of MEL's third parameter	-4.10
Variance of MEL's ninth parameter	2.36
Mean of MEL's eleventh parameter	1.81
Mean of MEL's first parameter	-1.73
Skewness of MEL's sixth parameter	0.44
Skewness of Root Mean Square	0.41
Variance of Onset Envelope	-0.28
Kurtosis of frequency bandwidth	-0.25
Skewness of MEL's eleventh parameter	0.24
Skewness of frequency bandwidth	-0.07
Variance of frequency bandwidth	-0.013
Mean Zero Crossing Rate	0.004

Note. MEL represents the spectral information of a voice signal using a vector of 13 parameters; SVM = Support Vector Machine.

2.1 Supplementary Figures

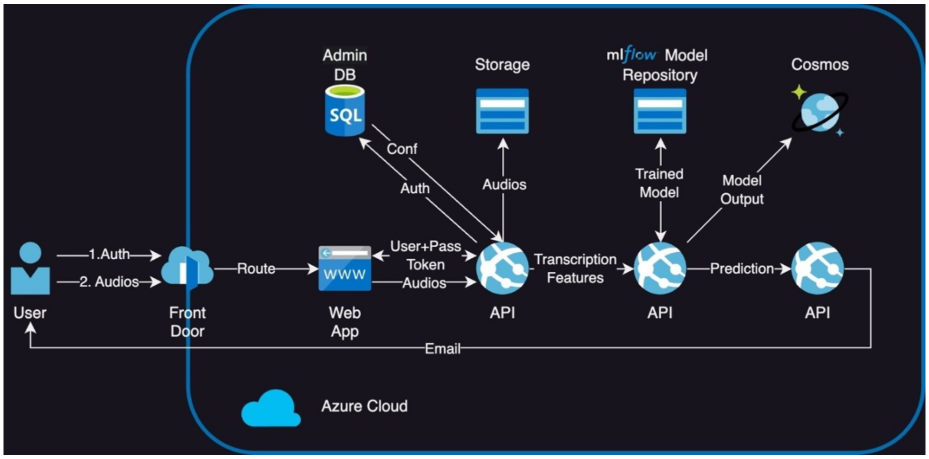

Supplementary Figure 1. Azure Cloud-Based Configuration Scheme of AcceXible Software.

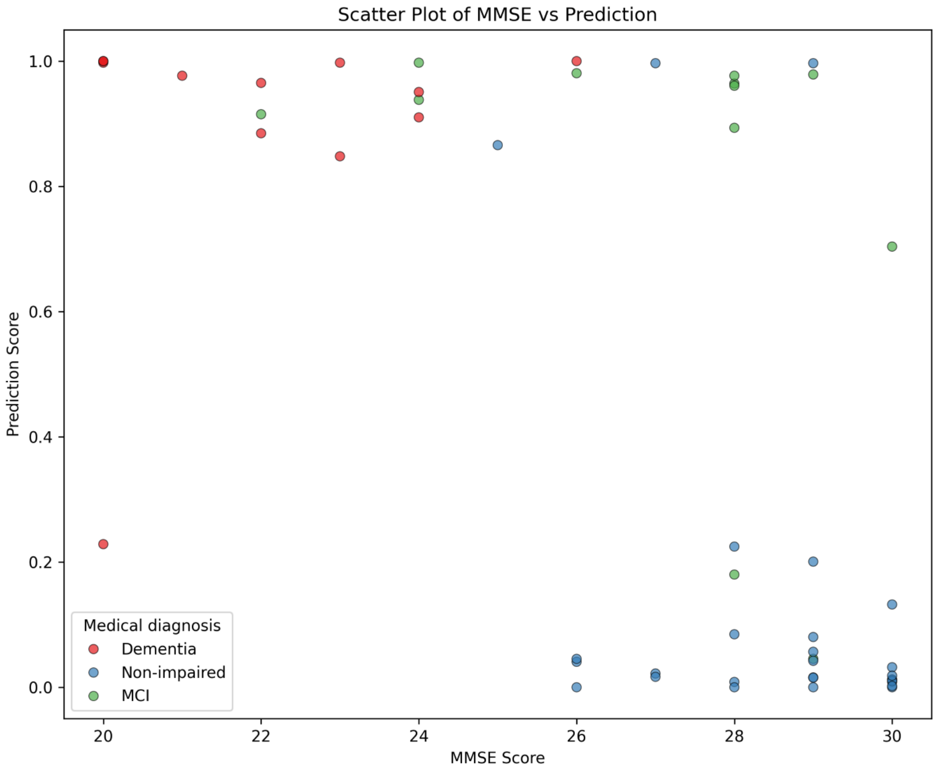

Supplementary Figure 2. Relationship between MMSE score and algorithm output in the testing set by diagnostic group: cognitively normal, mild cognitive impairment, and all-cause dementia.

References

Buitinck, L., Louppe, G., Blondel, M., Pedregosa, F., Mueller, A., Grisel, O., Niculae, V., Prettenhofer, P., Gramfort, A., Grobler, J., Layton, R., Vanderplas, J., Joly, A., Holt, B., & Varoquaux, G. (2013). *API design for machine learning software: Experiences from the scikit-learn project* (arXiv:1309.0238). arXiv. https://doi.org/10.48550/arXiv.1309.0238
